# Redetermined structure of 4,4′-bi­pyridine–1,4-phenyl­enedi­acetic acid (1/1) co-crystal

**DOI:** 10.1107/S2056989015017569

**Published:** 2015-09-26

**Authors:** Rima Paul, Sanchay Jyoti Bora

**Affiliations:** a Department of Chemistry, Pandu College, Guwahati-781 012, Assam, India

**Keywords:** crystal structure, co-crystal, supra­molecular inter­action, hydrogen bonding

## Abstract

The asymmetric unit of the title 1:1 co-crystal, C_10_H_8_N_2_·C_10_H_10_O_4_, consists of one half-mol­ecule each of 4,4′-bi­pyridine and 1,4-phenyl­enedi­acetic acid: the complete mol­ecules are generated by crystallographic inversion centres. The dihedral angle between the –CO_2_H group and the benzene ring in the diacid is 73.02 (7)°. In the crystal, the components are linked by O—H⋯N hydrogen bonds, generating [1-2-1] chains of alternating amine and carb­oxy­lic acid mol­ecules. The chains are cross-linked by C—H⋯O inter­actions. This structure was previously incorrectly described as a (C_10_H_10_N_2_)^2+^·(C_10_H_8_O_4_)^2−^ mol­ecular salt [Jia *et al.* (2009 ▸). *Acta Cryst.* E**65**, o2490–o2490].

## Related literature   

For the previous erroneous report of this structure as a mol­ecular salt, see: Jia *et al.* (2009 ▸). For hydrogen-bonded co-crystals, see: Stahly (2009 ▸); Kavuru *et al.* (2010 ▸). For pharmaceutical co-crystals, see: Childs *et al.* (2009 ▸); Walsh *et al.* (2003 ▸). For a similar structure, see: Chinnakali *et al.* (1999 ▸).
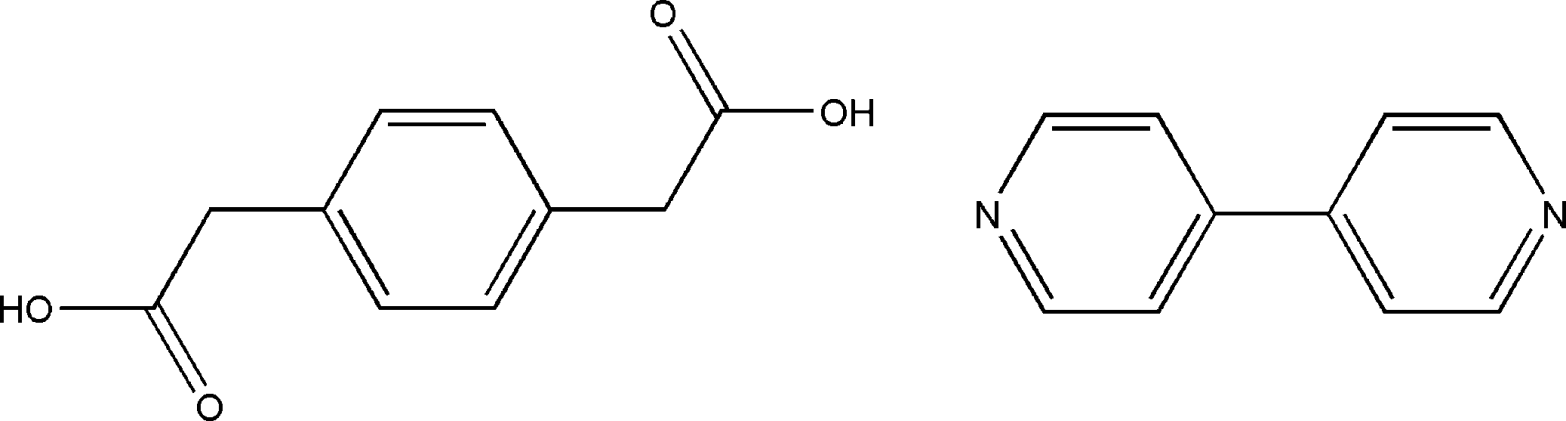


## Experimental   

### Crystal data  

C_10_H_8_N_2_·C_10_H_10_O_4_
*M* *_r_* = 350.36
Triclinic, 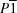

*a* = 4.5577 (5) Å
*b* = 6.9806 (8) Å
*c* = 13.7995 (15) Å
α = 99.508 (6)°
β = 94.297 (6)°
γ = 97.643 (7)°
*V* = 427.05 (8) Å^3^
*Z* = 1
Mo *K*α radiation
μ = 0.10 mm^−1^
*T* = 296 K
0.20 × 0.17 × 0.13 mm

### Data collection  

Bruker SMART CCD diffractometer
8581 measured reflections
2405 independent reflections
1876 reflections with *I* > 2σ(*I*)
*R*_int_ = 0.028

### Refinement  

*R*[*F* ^2^ > 2σ(*F* ^2^)] = 0.041
*wR*(*F* ^2^) = 0.123
*S* = 1.05
2405 reflections
155 parameters
All H-atom parameters refined
Δρ_max_ = 0.19 e Å^−3^
Δρ_min_ = −0.16 e Å^−3^

### 

Data collection: *SMART* (Bruker, 2004 ▸); cell refinement: *SAINT* (Bruker, 2004 ▸); data reduction: *SAINT*; program(s) used to solve structure: *SHELXS97* (Sheldrick, 2008 ▸); program(s) used to refine structure: *SHELXL97* (Sheldrick, 2008 ▸); molecular graphics: *ORTEP-3 for Windows* (Farrugia, 2012 ▸); software used to prepare material for publication: *SHELXL97*.

## Supplementary Material

Crystal structure: contains datablock(s) I, New_Global_Publ_Block. DOI: 10.1107/S2056989015017569/hb7506sup1.cif

Structure factors: contains datablock(s) I. DOI: 10.1107/S2056989015017569/hb7506Isup2.hkl

Supporting information file. DOI: 10.1107/S2056989015017569/hb7506Isup3.doc

**Fig. 1.**
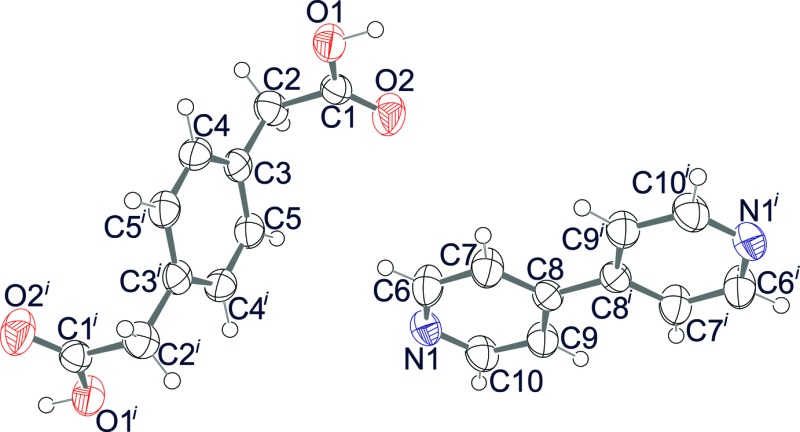
The molecular structure of (I) showing 50% probability displacement ellipsoids.

I . DOI: 10.1107/S2056989015017569/hb7506fig1.tif
The mol­ecular structure of (**I**) showing 50% probability displacement ellipsoids.

**Fig. 2.**
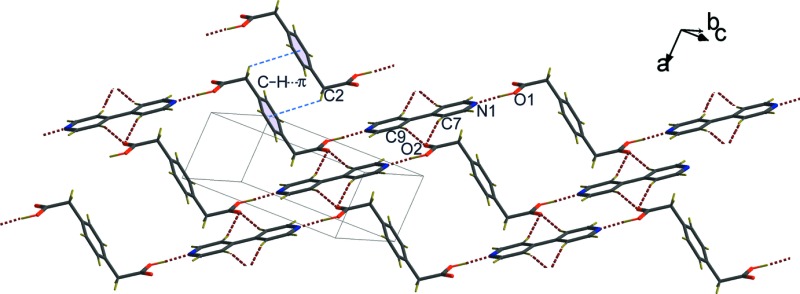
A view of the O—H···N, C—H···O and C—H···π interactions observed in the crystal structure of the title compound.

. DOI: 10.1107/S2056989015017569/hb7506fig2.tif
A view of the O—H⋯N, C—H⋯O and C—H⋯π inter­actions observed in the crystal structure of the title compound.

CCDC reference: 1423417

Additional supporting information:  crystallographic information; 3D view; checkCIF report

## Figures and Tables

**Table 1:** Hydrogen-bond geometry (, )

*D*H*A*	*D*H	H*A*	*D* *A*	*D*H*A*
O1H9N1^i^	1.02(2)	1.62(2)	2.6373(13)	176(2)
C7H6O2^ii^	0.954(16)	2.504(16)	3.4196(16)	160.8(11)
C9H7O2^iii^	1.00(2)	2.45(2)	3.4205(18)	162.2(16)

Symmetry codes: (i) 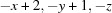; (ii) 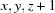; (iii) 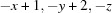.

## supplementary crystallographic information

### S1. Comment

Co-crystals represent a class of materials which contain two or more discrete molecular entities held together *via* non-covalent or supra­molecular inter­actions in the crystal lattice (Stahly, 2009). Due to their robust and directional nature, hydrogen bonds are extensively used as a tool to shape the structure of co-crystals (Kavuru *et al.*, 2010). In this context, hydrogen bonds of varying strengths may be employed, ranging from strong O—H···O/N to weak C—H···π inter­actions. The resulting crystal structures can generate diverse physical and chemical properties such as solubility and stability that differ from the properties of the individual components. Crystal engineering plays an important role in the formation of co-crystals of desired properties so that they can find their applications in pharmaceutical industries (Childs *et al.*, 2009 and Walsh *et al.*, 2003). Herein, we report the supra­molecular architecture of 1,4-phenyl­enedi­acetic acid and 4,4'-bi­pyridine co-crystal formed *via* O—H···N hydrogen bridges and C—H···π inter­actions.

The title compound can be prepared under hydro­thermal condition using a mixture of 1,4-phenyl­enedi­acetic acid and 4,4'-bi­pyridine (1:1) in water. The acetic acid moiety involving C1, C2, O1 and O2 in 1,4-phenyl­enedi­acetic acid molecule makes dihedral angles of 73.04 (4)° and 2.06 (1)° with the phenyl and pyridyl ring planes respectively. These values are very close to those reported by Chinnakali *et al.* (1999). The dihedral angle between phenyl and planar pyridyl rings of 4,4'-bi­pyridine is found to be 73.21 (4)°. In the crystal lattice, the molecules are linked with one another through O1—H9···N1 hydrogen bonds with O···N distance of 2.637 (1) Å that extends in one direction leading to a supra­molecular chain like structure. These zig-zag 1D chains are further connected via C—H···O bridges (C7—H6···O2 and C9—H7···O2 with C···O distances of 2.50 (1) Å and 2.45 (2) Å respectively) giving rise to a 2D layered structure in the solid state. In graph set notations (Bernstain *et al.*, 1995), such 1D chains can be described as *C*_2_^2^(20) where the subscripts and superscripts are the number of hydrogen bond donors and acceptors respectively. There are certain hydrogen bonded rings of descriptors *R*_1_^2^(7), *R*_4_^4^(16) and *R*_4_^4^(30) which have periodic repetitions throughout the crystal lattice. The adjacent layers are stacked in nearly parallel fashion by means of weak C—H···π inter­actions (C···π distance = 3.838 Å) between the methyl­ene C—H and phenyl ring–π systems. These weak inter­molecular forces together with the strong hydrogen bonds form the overall 3D supra­molecular architecture.

?

### S2. Synthesis and crystallization

A mixture of 1,4-phenyl­enedi­acetic acid (1 mmol, 0.194 g) and 4,4?-bi­pyridine (1 mmol, 0.156 g) in water (10 ml) were placed in a 23 ml Teflon lined stainless steel reaction vessel. It was then heated to 393K for 24 hours at a heating rate of 5K min^-1^. On overnight standing, re­cta­ngular block shaped colourless crystals were obtained. The crystals were then filtered off, washed with water and dried in a vacuum desiccator over fused CaCl_2_. Yield: 71%.

### S3. Refinement

Structure determination work was done using the *WinGX* platform (Farrugia, 2012). All the hydrogen atoms were located in difference Fourier maps and refined with isotropic atomic displacement parameters. No restraints were applied for any other parameter during structure refinement.

### Crystal data

**Table:**

C_10_H_8_N_2_·C_10_H_10_O_4_	*V* = 427.05 (8) Å^3^
*M**_r_* = 350.36	*Z* = 1
Triclinic, *P*1	*F*(000) = 184
*a* = 4.5577 (5) Å	*D*_x_ = 1.362 Mg m^−^^3^
*b* = 6.9806 (8) Å	Mo *K*α radiation, λ = 0.71073 Å
*c* = 13.7995 (15) Å	µ = 0.10 mm^−^^1^
α = 99.508 (6)°	*T* = 296 K
β = 94.297 (6)°	Rectangular block, colourless
γ = 97.643 (7)°	0.20 × 0.17 × 0.13 mm

### Data collection

**Table:**

Bruker SMART CCD diffractometer	1876 reflections with *I* > 2σ(*I*)
Radiation source: fine-focus sealed tube	*R*_int_ = 0.028
Graphite monochromator	θ_max_ = 29.8°, θ_min_ = 3.0°
phi and ω scans	*h* = −6→6
8581 measured reflections	*k* = −9→9
2405 independent reflections	*l* = −19→19

### Refinement

**Table:**

Refinement on *F*^2^	Secondary atom site location: difference Fourier map
Least-squares matrix: full	Hydrogen site location: inferred from neighbouring sites
*R*[*F*^2^ > 2σ(*F*^2^)] = 0.041	All H-atom parameters refined
*wR*(*F*^2^) = 0.123	*w* = 1/[σ^2^(*F*_o_^2^) + (0.0605*P*)^2^ + 0.0515*P*] where *P* = (*F*_o_^2^ + 2*F*_c_^2^)/3
*S* = 1.05	(Δ/σ)_max_ = 0.001
2405 reflections	Δρ_max_ = 0.19 e Å^−^^3^
155 parameters	Δρ_min_ = −0.16 e Å^−^^3^
0 restraints	Extinction correction: *SHELXL*, Fc^*^=kFc[1+0.001xFc^2^λ^3^/sin(2θ)]^-1/4^
Primary atom site location: structure-invariant direct methods	Extinction coefficient: 0.061 (11)

### Special details

**Table:**

Geometry. All s.u.'s (except the s.u. in the dihedral angle between two l.s. planes) are estimated using the full covariance matrix. The cell s.u.'s are taken into account individually in the estimation of s.u.'s in distances, angles and torsion angles; correlations between s.u.'s in cell parameters are only used when they are defined by crystal symmetry. An approximate (isotropic) treatment of cell s.u.'s is used for estimating s.u.'s involving l.s. planes.
Refinement. Refinement of *F*^2^ against ALL reflections. The weighted *R*-factor *wR* and goodness of fit *S* are based on *F*^2^, conventional *R*-factors *R* are based on *F*, with *F* set to zero for negative *F*^2^. The threshold expression of *F*^2^ > 2σ(*F*^2^) is used only for calculating *R*-factors(gt) *etc*. and is not relevant to the choice of reflections for refinement. *R*-factors based on *F*^2^ are statistically about twice as large as those based on *F*, and *R*- factors based on ALL data will be even larger.

### Fractional atomic coordinates and isotropic or equivalent isotropic displacement parameters (Å^2^)

**Table:**

	*x*	*y*	*z*	*U*_iso_*/*U*_eq_	
O1	0.8197 (2)	0.45294 (13)	−0.18830 (6)	0.0541 (3)	
C1	0.7209 (2)	0.60720 (15)	−0.21279 (8)	0.0408 (3)	
C2	0.5619 (3)	0.71533 (19)	−0.13310 (10)	0.0490 (3)	
C3	0.7850 (2)	0.86342 (15)	−0.06291 (8)	0.0400 (3)	
C4	0.9459 (3)	0.80852 (16)	0.01476 (9)	0.0454 (3)	
C5	0.8422 (3)	1.05717 (17)	−0.07687 (8)	0.0446 (3)	
O2	0.7616 (3)	0.66207 (15)	−0.28973 (7)	0.0675 (3)	
N1	0.8832 (2)	0.73182 (15)	0.32203 (7)	0.0476 (3)	
C6	0.8316 (3)	0.67172 (18)	0.40629 (9)	0.0517 (3)	
C7	0.6844 (3)	0.77149 (18)	0.47798 (9)	0.0487 (3)	
C8	0.5796 (2)	0.94340 (15)	0.46266 (7)	0.0376 (2)	
C10	0.7840 (3)	0.8964 (2)	0.30711 (10)	0.0566 (3)	
C9	0.6326 (3)	1.00451 (19)	0.37423 (9)	0.0532 (3)	
H9	0.927 (5)	0.382 (3)	−0.2421 (15)	0.104 (7)*	
H3	0.908 (3)	0.676 (2)	0.0263 (11)	0.061 (4)*	
H4	0.731 (3)	1.100 (2)	−0.1302 (11)	0.055 (4)*	
H6	0.660 (3)	0.722 (2)	0.5378 (12)	0.067 (4)*	
H1	0.462 (4)	0.621 (2)	−0.0977 (12)	0.065 (4)*	
H8	0.822 (4)	0.935 (2)	0.2459 (13)	0.073 (5)*	
H7	0.560 (4)	1.122 (3)	0.3533 (13)	0.081 (5)*	
H2	0.412 (4)	0.787 (3)	−0.1669 (12)	0.071 (5)*	
H5	0.902 (4)	0.551 (3)	0.4159 (12)	0.071 (4)*	

### Atomic displacement parameters (Å^2^)

**Table:**

	*U*^11^	*U*^22^	*U*^33^	*U*^12^	*U*^13^	*U*^23^
O1	0.0764 (6)	0.0483 (5)	0.0463 (5)	0.0272 (4)	0.0173 (4)	0.0141 (4)
C1	0.0449 (6)	0.0370 (5)	0.0403 (5)	0.0071 (4)	0.0035 (4)	0.0055 (4)
C2	0.0434 (6)	0.0489 (6)	0.0549 (7)	0.0115 (5)	0.0119 (5)	0.0025 (5)
C3	0.0431 (5)	0.0405 (5)	0.0396 (5)	0.0148 (4)	0.0163 (4)	0.0046 (4)
C4	0.0580 (7)	0.0363 (5)	0.0467 (6)	0.0141 (5)	0.0146 (5)	0.0116 (4)
C5	0.0545 (7)	0.0452 (6)	0.0394 (5)	0.0184 (5)	0.0097 (5)	0.0116 (4)
O2	0.1023 (8)	0.0630 (6)	0.0502 (5)	0.0351 (6)	0.0215 (5)	0.0232 (4)
N1	0.0507 (6)	0.0478 (5)	0.0450 (5)	0.0158 (4)	0.0075 (4)	0.0023 (4)
C6	0.0643 (8)	0.0439 (6)	0.0511 (7)	0.0221 (6)	0.0096 (6)	0.0075 (5)
C7	0.0631 (7)	0.0441 (6)	0.0443 (6)	0.0189 (5)	0.0116 (5)	0.0117 (5)
C8	0.0370 (5)	0.0374 (5)	0.0379 (5)	0.0073 (4)	0.0026 (4)	0.0045 (4)
C10	0.0714 (9)	0.0614 (8)	0.0466 (7)	0.0285 (6)	0.0200 (6)	0.0159 (6)
C9	0.0691 (8)	0.0515 (7)	0.0490 (6)	0.0284 (6)	0.0181 (6)	0.0166 (5)

### Geometric parameters (Å, º)

**Table:**

O1—C1	1.3051 (13)	C5—H4	0.972 (14)
O1—H9	1.02 (2)	N1—C6	1.3264 (16)
C1—O2	1.2056 (14)	N1—C10	1.3301 (16)
C1—C2	1.5147 (16)	C6—C7	1.3820 (17)
C2—C3	1.5108 (17)	C6—H5	0.964 (17)
C2—H1	0.970 (16)	C7—C8	1.3906 (15)
C2—H2	1.027 (17)	C7—H6	0.955 (16)
C3—C4	1.3877 (16)	C8—C9	1.3850 (16)
C3—C5	1.3908 (15)	C8—C8^ii^	1.486 (2)
C4—C5^i^	1.3844 (18)	C10—C9	1.3810 (17)
C4—H3	0.961 (15)	C10—H8	0.950 (18)
C5—C4^i^	1.3844 (18)	C9—H7	0.998 (19)
			
C1—O1—H9	112.6 (12)	C3—C5—H4	120.1 (8)
O2—C1—O1	123.26 (10)	C6—N1—C10	117.10 (10)
O2—C1—C2	123.39 (11)	N1—C6—C7	123.47 (11)
O1—C1—C2	113.30 (10)	N1—C6—H5	116.3 (10)
C3—C2—C1	109.58 (9)	C7—C6—H5	120.2 (10)
C3—C2—H1	110.0 (9)	C6—C7—C8	119.66 (11)
C1—C2—H1	108.9 (10)	C6—C7—H6	119.0 (10)
C3—C2—H2	109.4 (9)	C8—C7—H6	121.4 (10)
C1—C2—H2	107.7 (9)	C9—C8—C7	116.53 (10)
H1—C2—H2	111.2 (13)	C9—C8—C8^ii^	121.59 (12)
C4—C3—C5	118.28 (11)	C7—C8—C8^ii^	121.88 (12)
C4—C3—C2	121.06 (10)	N1—C10—C9	123.35 (12)
C5—C3—C2	120.61 (10)	N1—C10—H8	115.3 (10)
C5^i^—C4—C3	120.99 (10)	C9—C10—H8	121.3 (10)
C5^i^—C4—H3	119.3 (9)	C10—C9—C8	119.89 (11)
C3—C4—H3	119.7 (9)	C10—C9—H7	116.0 (11)
C4^i^—C5—C3	120.73 (11)	C8—C9—H7	124.1 (11)
C4^i^—C5—H4	119.2 (8)		

Symmetry codes: (i) −*x*+2, −*y*+2, −*z*; (ii) −*x*+1, −*y*+2, −*z*+1.

### Hydrogen-bond geometry (Å, º)

**Table:**

*D*—H···*A*	*D*—H	H···*A*	*D*···*A*	*D*—H···*A*
O1—H9···N1^iii^	1.02 (2)	1.62 (2)	2.6373 (13)	176 (2)
C7—H6···O2^iv^	0.954 (16)	2.504 (16)	3.4196 (16)	160.8 (11)
C9—H7···O2^v^	1.00 (2)	2.45 (2)	3.4205 (18)	162.2 (16)

Symmetry codes: (iii) −*x*+2, −*y*+1, −*z*; (iv) *x*, *y*, *z*+1; (v) −*x*+1, −*y*+2, −*z*.
