# Supplementary material for: Redetermined structure of 4,4′-bi­pyridine–1,4-phenyl­enedi­acetic acid (1/1) co-crystal
Source: Acta Crystallogr E Crystallogr Commun. 2015 Sep 26;71(Pt 10):o799–800. doi: 10.1107/S2056989015017569 (PMC4647443; doi:10.1107/S2056989015017569)
Supplement: Supplementary file 3 [file e-71-0o799-Isup3.doc]

**1,4-phenylenediacetic acid and 4,4'-bipyridine (1:1) co-crystal**

**Rima Paul, Sanchay Jyoti Bora**

1. **Comment**

Co-crystals represent a class of materials which contain two or more discrete molecular entities held together *via* non-covalent or supramolecular interactions in the crystal lattice (Stahly, 2009). Due to their robust and directional nature, hydrogen bonds are extensively used as a tool to shape the structure of co-crystals (Kavuru *et al*., 2010). In this context, hydrogen bonds of varying strengths may be employed, ranging from strong O−H⋅⋅⋅O/N to weak C−H⋅⋅⋅π interactions. The resulting crystal structures can generate diverse physical and chemical properties such as solubility and stability that differ from the properties of the individual components. Crystal engineering plays an important role in the formation of co-crystals of desired properties so that they can find their applications in pharmaceutical industries (Childs *et al*., 2009 and Walsh *et al*., 2003). Herein, we report the supramolecular architecture of 1,4-phenylenediacetic acid and 4,4’-bipyridine co-crystal formed *via* O−H⋅⋅⋅N hydrogen bridges and C−H⋅⋅⋅π interactions.

The title compound can be prepared under hydrothermal condition using a mixture of 1,4-phenylenediacetic acid and 4,4’-bipyridine (1:1) in water. The acetic acid moiety involving C1, C2, O1 and O2 in 1,4-pheylenediacetic acid molecule makes dihedral angles of 73.04(4)º and 2.06(1)º with the phenyl and pyridyl ring planes respectively. These values are very close to those reported by Chinnakali *et al*. (1999). The dihedral angle between phenyl and planer pyridyl rings of 4,4’-bipyridine is found to be 73.21(4)º. In the crystal lattice, the molecules are linked with one another through O1−H9⋅⋅⋅N1 hydrogen bonds with O⋅⋅⋅N distance of 2.637(1) Å that extends in one direction leading to a supramolecular chain like structure. These zig-zag 1D chains are further connected via C−H⋅⋅⋅O bridges (C7−H6⋅⋅⋅O2 and C9−H7⋅⋅⋅O2 with C⋅⋅⋅O distances of 2.50(1) Å and 2.45(2) Å respectively) giving rise to a 2D layered structure in the solid state. In graph set notations (Bernstain *et al.*, 1995), such 1D chains can be described as
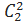
(20) where the subscripts and superscripts are the number of hydrogen bond donors and acceptors respectively. There are certain hydrogen bonded rings of descriptors
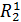
(7),
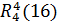
 and
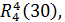
which have periodic repetitions throughout the crystal lattice. The adjacent layers are stacked in nearly parallel fashion by means of weak C−H⋅⋅⋅π interactions (C⋅⋅⋅π distance = 3.838 Å) between the methylene C−H and phenyl ring-π systems. These weak intermolecular forces together with the strong hydrogen bonds form the overall 3D supramolecular architecture.

1. **Experimental**

A mixture of 1,4-phenylenediacetic acid (1 mmol, 0.194 g) and 4,4’-bipyridine (1 mmol, 0.156 g) in water (10 ml) were placed in a 23ml Teflon lined stainless steel reaction vessel. It was then heated to 393K for 24 hours at a heating rate of 5K min^-1^. On overnight standing, rectangular block shaped colourless crystals were obtained. The crystals were then filtered off, washed with water and dried in a vacuum desiccator over fused CaCl_2_. Yield: 71%.

1. **Refinement**

Structure determination work was done using the WinGX platform (Farrugia, 1999). All the hydrogen atoms were located in difference Fourier maps and refined with isotropic atomic displacement parameters. No restraints were applied for any other parameter during structure refinement.
