# Supplementary figures and images for: Redetermined structure of 4,4′-bi­pyridine–1,4-phenyl­enedi­acetic acid (1/1) co-crystal
Source: Acta Crystallogr E Crystallogr Commun. 2015 Sep 26;71(Pt 10):o799–800. doi: 10.1107/S2056989015017569 (PMC4647443; doi:10.1107/S2056989015017569)

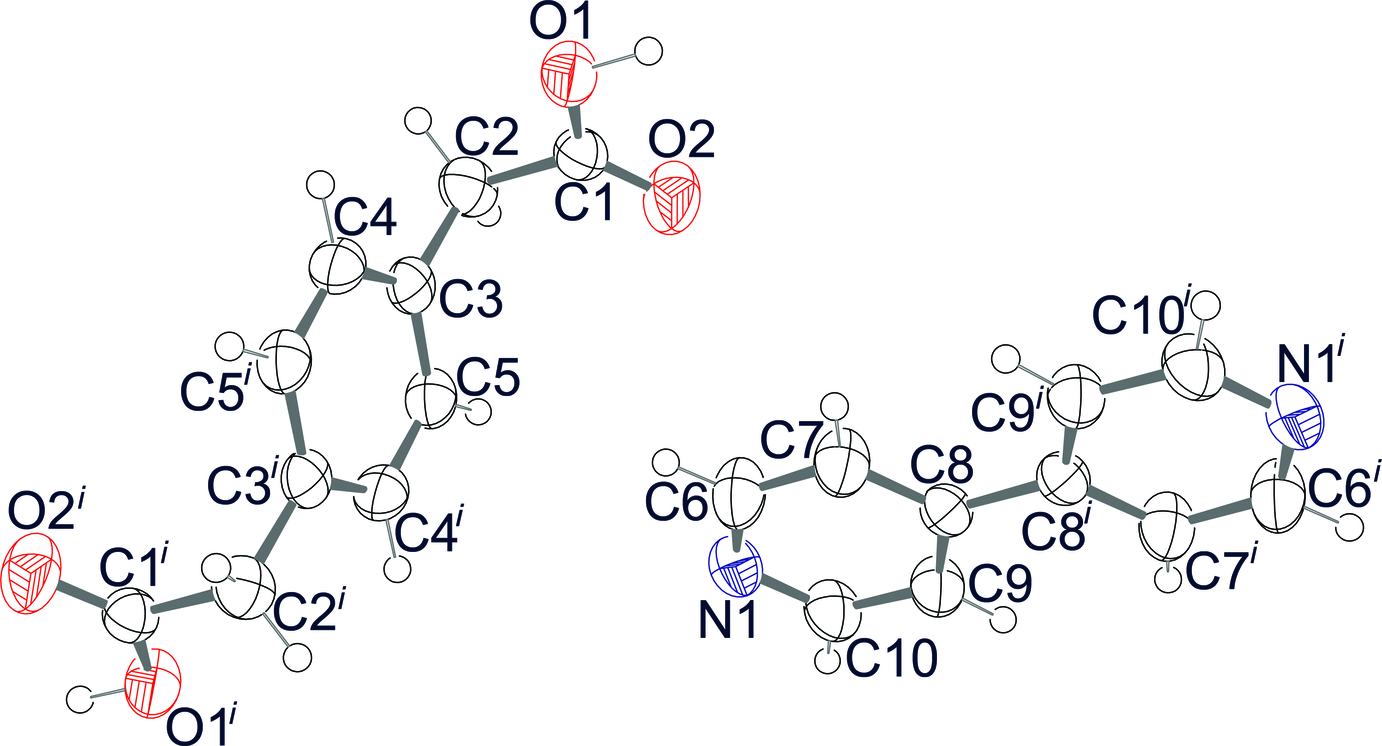

Supplement: Supplementary file 4 [file e-71-0o799-fig1.tif]

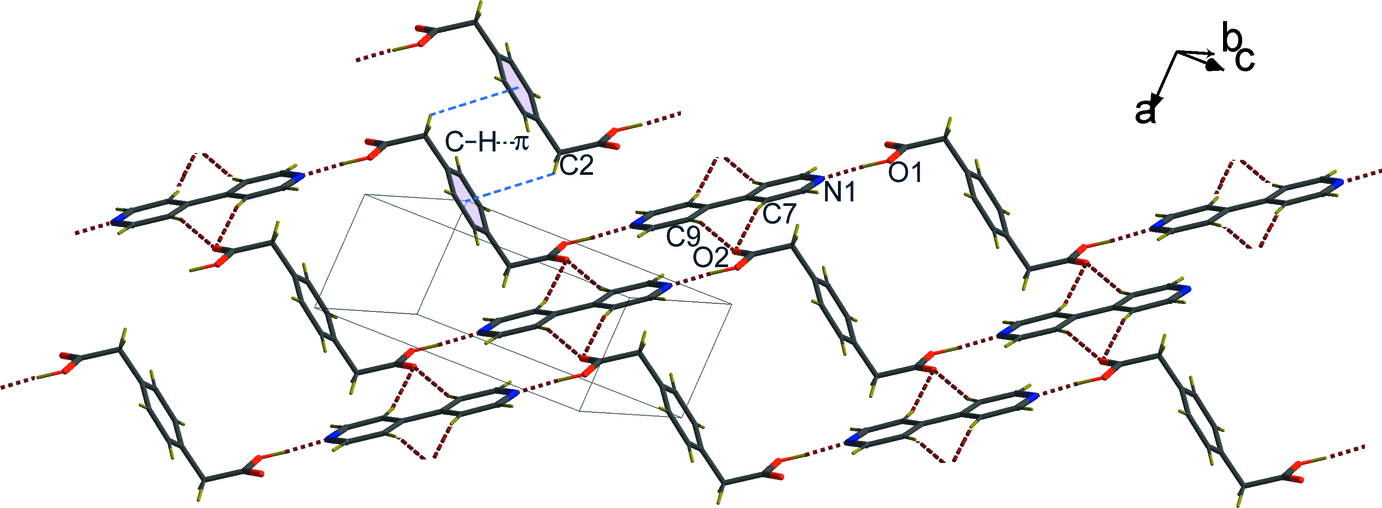

Supplement: Supplementary file 5 [file e-71-0o799-fig2.tif]
